# Patient and public involvement in data collection for health services research: a descriptive study

**DOI:** 10.1186/s40900-015-0006-7

**Published:** 2015-08-07

**Authors:** Sara Garfield, Seetal Jheeta, Ann Jacklin, Anna Bischler, Christine Norton, Bryony D. Franklin

**Affiliations:** 1 Centre for Medication Safety and Service Quality, Imperial College Healthcare NHS Trust, London, UK; 2 The Department of Practice and Policy, UCL School of Pharmacy, Mezzanine Floor, BMA House, Tavistock Square, London, UK; 3 Pharmacy Department, Chelsea and Westminster Healthcare NHS Foundation Trust, London, UK; 4 King’s College London, Faculty of Nursing & Midwifery & Imperial College Healthcare NHS Trust, 57 Waterloo Road, London, SE1 8WA USA

**Keywords:** Patient and public involvement, Lay involvement, Observation, Data collection, Hospitals, Health services research

## Abstract

**Plain English summary:**

There is a consensus that patients and the public should be involved in research in a meaningful way. To date, lay people have been mostly involved in developing research ideas and commenting on patient information but not as much in actual data collection.

We have had firsthand experience with lay people helping to conduct a study on how patients in hospital are involved with their medicines. In the first part of this study, we observed doctors’ ward rounds, pharmacists’ ward visits and nurses’ drug administration rounds, to find out if and how healthcare professionals interacted with patients about their medicines. Lay people conducted some of these observations. We wanted to explore the benefits and challenges of having lay people conduct these observations, to tell us more about how lay people can be involved in conducting such research.

We interviewed the lay members and researchers involved in this research to find out their views. We also looked at the observation notes to identify what the lay people had noticed that the researchers had not.

The lay members and researchers reported that lay members added value to the study by bringing new perspectives. Lay people had noticed some different things to the researchers. We experienced some challenges which need to be addressed. These were

- getting the lay observers registered with the hospitals to allow them to be on the wards in this capacity
- lay observers and researchers having different understanding of research procedures such as patient consent
- trying to find lay observers of different backgrounds and ethnic groups

**Abstract:**

**Background:** It is recognised that involving lay people with research in a meaningful rather than tokenistic way is both important and challenging. In a recent health services research study addressing inpatient involvement in medication safety, we sought to overcome this challenge by including lay people in collecting observational data in the hospital setting. The aim of this study was to evaluate lay and researcher perspectives on lay involvement in data collection in order to inform and enhance the future role of lay people in carrying out health services research.

**Methods:** We conducted semi-structured interviews with the lay members who collected observational data in our wider study and the researchers who provided support and/or were involved in their recruitment and training. The interviews were transcribed verbatim and coded using open thematic analysis. In addition, we conducted secondary analysis of the observational data to identify the specific contributions of lay observers.

**Results:** We interviewed the three lay members and the four researchers involved. Both these interviews and the secondary data analysis demonstrated that the lay members added value to the study by bringing additional general perspectives on communication with hospital inpatients.

Combined with researchers’ perspectives on interactions more specifically related to medication, this provided a broader answer to our research question of how healthcare professionals facilitate inpatient understanding of their medication and involvement in medication safety. This contrasted to the involvement lay observers reported having in previous research where their role had been more consultative. The lay members all reported that carrying out the observations had been an interesting and informative experience.

Some challenges arose including the infrastructure not having been in place to support this specialist lay research role, differing paradigms of research governance held by the public and researchers in relation to consent procedures and difficulties in recruiting a diverse range of members of the public to carry out the role.

**Conclusions:** Lay members can add value to research by being involved in data collection within health services research. There is a need to build infrastructure to better support this involvement.

## Background

There has been increased recognition of the potential benefits of patient and public involvement in research, and researchers are increasingly being asked to demonstrate patient involvement to funding bodies [1]. Suggested benefits include improved quality of the results, more direct applicability of research to patients and improved translation into clinical practice [2]. Lay involvement has also been seen as ethically mandatory [2].

However, there is also concern that current patient and public involvement is often tokenistic and aimed at ‘ticking boxes for funding applications’, rather than placing a true value on patient input and involvement [1, 2]. Domecq et al. [2] carried out a systematic review of studies which described involving patients in the conduct, design and/or dissemination of research. They found that lay people were mostly involved in agenda setting and protocol development, and it was much less common for lay people to be involved in execution or translation of the research; only a very small number of studies involved lay people in data collection. Evaluation of different approaches to involving patients and the public is required [1, 2].

The extent to which lay members can carry out these more extensive research roles has been debated. For example, Ives [3] and Martin and Finn [4] have discussed whether it is possible to give lay people the training required to conduct research without turning them into ‘professional’ researchers and losing their unique lay perspective. There has also been discussion about the best way of achieving representation in terms of lay involvement [5].

We have recent experience of involving lay people in a health services research study that has seen their roles extend beyond agenda setting and protocol development and into data collection. The study explored how inpatients can be involved in medication safety while in hospital and the potential role of electronic prescribing systems as a barrier or facilitator (the Inpatient Medication, Patient Relationships and Electronic SystemS (IMPRESS) Study [6]). The first phase of this study involved carrying out observations of doctors’ ward rounds, pharmacists’ ward visits and nurses’ drug administration rounds in two English National Health Service (NHS) hospital trusts (organisations), to explore if and how healthcare professionals facilitate inpatient involvement in medication safety and how this may differ between paper and electronic systems. The project had a patient and clinical engagement group that met quarterly and originally included three lay members and relevant healthcare professionals. At the first meeting of this group, one of the lay members expressed the view that our proposed patient and public involvement was not sufficient to truly include lay perspectives in the research. The suggestions were put forward that additional lay people should be recruited to the patient and clinical engagement group and that lay people should be involved in conducting some of the observations; these suggestions were adopted.

The aim of the present paper is to describe our experiences of lay involvement in conducting research, from both the lay observers’ and researchers’ perspectives, in order to inform the future role of lay people in carrying out health services research. We describe experiences of the preparation for the lay involvement, the lay observations themselves, the value added by involving lay observers in data collection and the challenges that arose.

## Methods

### Setting

The IMPRESS study took place at two acute London hospital trusts. Three lay observers carried out observations in one trust, and one of these lay observers also carried out observations in the second. This was due to more extensive procedures for registering lay observers at the second trust, as described below.

### Ethics

An ethics amendment was submitted to the ethics committee that had approved the original IMPRESS study to request approval to also include lay observers. The proposed amendment stated that the researchers, rather than lay observers, would invite informed consent from potential participants and would be present on the ward while lay observers were conducting observations to act as a support. This amendment was approved subject to the requirement that all lay observers had a disclosure and barring service (DBS) check and were covered by suitable indemnity insurance.

### Training for lay observers

We registered the lay observers as hospital volunteers as the volunteer registration process included a DBS check and registered volunteers were covered by appropriate insurance. At both trusts, the lay observers received the standard in-house training for hospital volunteers. In addition, the IMPRESS research team led a training session on the study’s data collection procedures; this included conduct of the observations, the relevant information and research governance procedures and the general scope of the observations. We did not include very specific training on the data to be collected, in order to provide sufficient opportunity for lay observers to bring their own perspectives to the observations. The processes in place for hospital volunteers meant that the lay observers had to come in on separate occasions for the DBS check and the volunteer training at each trust. At one of the trusts, a third appointment was also required for an occupational health check.

### Procedure for carrying out observations

Prior to the lay observations, the researchers distributed information leaflets to all patients and healthcare professionals who were likely to be involved in the relevant healthcare professional round being observed. The researchers then invited verbal consent from these patients and healthcare professionals, as required by the ethics committee.

For observations of doctors’ ward rounds, which typically include a whole team of healthcare professionals, the lay observer was present together with one of the research team observers. The two observers attended the round together taking independent notes. However, for observations of pharmacists’ ward rounds and nurses’ drug administration rounds where only one healthcare professional was being observed at each time, we were concerned that being observed by two people would feel intimidating. For these rounds, a researcher therefore obtained consent from the relevant healthcare professionals and patients and was present on the ward at the time of the observations, but the lay observers conducted these observations independently. The lay observers carried out all observations on a voluntary basis.

In total, lay observers collected data relating to healthcare professionals’ interactions with 41 patients who consented to take part in the study.

### Evaluation

In July and August 2014, we conducted semi-structured interviews to explore the perspectives of the three lay observers and four researchers who were involved in the lay observations. Topics explored included previous experience of patient and public involvement in research, experiences of recruitment and training for the IMPRESS study, experiences of lay observations, benefits, challenges and lessons learnt for future studies. In addition, we conducted secondary analysis of the observational data to identify the specific contributions of the lay observers. NHS ethics approval was in place for the IMPRESS study but was not required to conduct these additional interviews, as the three lay observers were not recruited via the NHS.

### Recruitment and data collection

The lead IMPRESS researcher emailed the three lay observers who had conducted observations and the three other research team members involved in their recruitment, training or support, to invite them to be interviewed at a mutually convenient time and location. The interviews were held face to face using a semi-structured interview schedule. With consent of the respondents, the interviews were audio recorded. The lead IMPRESS researcher conducted the interviews with the three lay observers and the other three research team members, one of whom in turn interviewed the lead researcher.

### Analysis

Analysis of interviews with lay observers and researchers: Interviews were transcribed verbatim and coded using an open thematic approach [7]. QSR Nvivo 8 was used to aid in this process. Two researchers independently coded the interviews, and consensus on all discrepancies was reached.

Secondary analysis of IMPRESS observational data: Observation data from the researchers and lay observers had previously been coded using thematic analysis, again using QSR Nvivo 8. For the present analysis, the researcher specifically identified the codes that appeared in the lay observation sources but not the researchers’ sources.

## Results

The three lay members (representing both genders) and four researchers all agreed to take part and were interviewed. The duration of the interviews ranged from 10 to 45 min. Verbatim quotes are used to illustrate themes.

### Recruitment of lay observers

Recruitment of lay observers was reported to be challenging. The patient and clinical engagement group originally suggested that additional lay members could be recruited from among existing registered hospital volunteers at the two trusts at which observations were to be carried out. However, the volunteer manager at one of the trusts reported that existing volunteers already had their own roles at the hospitals and did not have extra capacity, and at the second trust, there was little interest in response to an advertisement to existing volunteers.

I probably thought it would be a lot easier. I think when we first met in our initial meetings we were discussing that we needed lay people, I thought it would be quite easy because I knew we had volunteer… kind of hub, …I thought it would be quite easy … And then I guess I was surprised that [name of volunteer manager] couldn’t really kind of magic up someone quite quickly who would be keen to do that kind of thing. (Researcher 3)

Following this, recruitment was based on using existing contacts who had been involved in other patient and public involvement activities and two further lay members were recruited. Three of five members of the expanded patient and clinical engagement group conducted observations and a fourth expressed an interest and registered as a volunteer but was then not able to commit the time to carrying out observations due to changes in personal circumstances

### Previous experience of patient and public involvement

The lay observers and researchers all reported previous experience of being involved in research as lay people. One of the researchers had previous experience of lay people being involved as part of an ethics committee, and the other three had experience of lay people having a consultative role on study steering groups. The previous experience of the lay observers ranged from being a participant in a focus group to having a patient and public involvement consultative role on several research steering groups.

From both the researchers’ and lay observers’ perspectives, previous involvement in research had been much more limited than in the current project.

I think there are projects we wanted to involve lay people but they’d never – they’ve not been involved to any significant extent. It’s kind of like, “Oh we’ve got to put in this grant application, it’s got patient involvement as one of the criteria, we’d better try and do something” rather than really thinking the way we did in this project about how the lay observers were actually going to contribute to the project. It was more about ticking a box to get the grant application. (Researcher 2)

Well, very often when patients, patient reps are involved in research projects and initiatives and programmes they are basically consulted. Consultation can cover many things, can cover reading patient information, patient leaflets, reading such proposal in some cases adding in fact the dimension that the researchers may not have seen. It’s basically looking at something which has been already thought about, already at a stage of more or less the design has started and just asking the patients to have a look at it and express their views. (Lay observer 2).

### Other experiences lay observers brought to the project

The three lay observers reported different experiences that they were able to bring to the project; one had been a patient herself in hospital many times with multiple chronic conditions, one had experience of communication management and the third had experience working a physiotherapist and in educational environments in the past.

### Benefits of including lay observations

#### Positive experience for lay observers and their role in research

All three lay observers described conducting the observations as an enjoyable and interesting experience. One of the lay observers also expressed the view that the lay observers had an equal relationship with the researchers. Three of the researchers identified benefits to the research that subsequently resulted from these positive experiences. Two of these researchers expressed the view that being involved in the data collection had resulted in increased lay observers’ motivation and involvement with the project. The third was of the opinion that having the lay people play such an integral part in the research may also benefit any other research projects they were involved in, as they would have an increased understanding of the research process.

The bottom line is, as I say, I really enjoyed it and I found it really interesting and quite eye opening. I think it’s a really valuable experience. (Lay observer 1)

It was at corporate level playing field there is no differences basically a group of people who want to produce some improvement in a particular service area, healthcare and who want to join forces and a very co-operative way and the relationship between the healthcare professionals and the patient representatives is really excellent. (Lay observer 3)

It has… probably contributed to how involved that they’ll be later in the project and discussing our findings and their findings … has meant that their involvement isn’t token. It’s probably increased their motivation and interest as well so that will be useful. (Researcher 2)

#### Different perspectives

The two researchers who were most involved in conducting the observations and working with the lay observers were of the opinion that the lay observers had greatly contributed to the research findings by bringing different perspectives to those of the researchers; all three lay observers also expressed this view. While the researchers reported being focused on discussions related to medication, the lay observers brought a wider perspective of general communication and engagement management and the impact that this may have on medication-related discussions between healthcare professionals and patients.

Because I am far less knowledgeable about medication and prescribing than the investigators I was working with, I concentrated much more on the human relationship between the inpatient, the patient in the bed and the consultant … when it was a consultant led ward round, or the pharmacist on a pharmacist ward round or the nurse/nursing student on a nurse ward round, the interaction, the body language, the relationship, the physical interaction, physical location and placement [of] the healthcare professional in relation to the patient in the bed. (Lay observer 2)

I felt the lay observers were picking up more about the overall conversation, about how the patient was involved, and not specific to medication-related issues, it was more of a general “how’s the patient being involved in decision making and in the conversation”, that kind of thing. So they definitely brought a different perspective, so it was really positive, it’s a really positive thing and it almost made me reflect how I was looking at the situation as well. (Researcher 1)

Secondary analysis of the IMPRESS data demonstrated that there were additional codes added to the coding framework specifically as a result of the lay observers’ involvement. These comprised length of consultation, consultation structure, communication style, the level of engagement of the patient and the lay perception of the patient situation. The secondary analysis also suggested that lay observers of different backgrounds brought different perspectives. For example, the lay observer who had a background in communication and customer management brought a fresh perspective on the effect that the consultation structure and communication style of the healthcare professional team might have on patient involvement in medication decisions and the lay observer who had his/her own experience as an inpatient documented observations based on empathy with the inpatients’ perspectives.

**Fig. 1:**
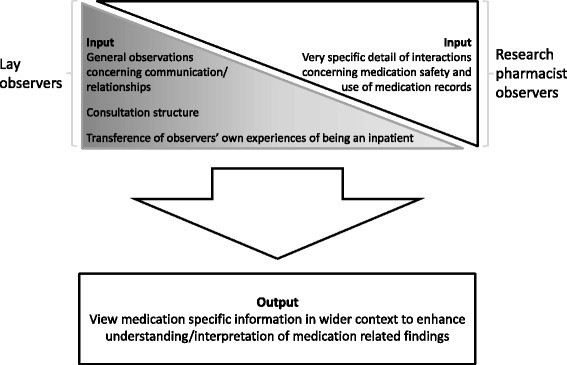
Distribution of observational input between lay and research pharmacist observers

Overall, interviews and secondary analysis suggested that combining the lay and researcher perspectives together gave a richer output than either could have given alone (Fig. 1). For example, the researchers reported observing that some discussions about medication that happen on doctors’ ward rounds did not involve the patient. The lay observers noted that ward round consultations were structured so that there was a ‘technical phase’ where the healthcare professional discussed the patient, in which the patient was about ‘as involved as a coffee pot’ followed by a ‘summary phase’ to the patient (coded as consultation structure). Taking the lay and researcher perspectives together, it became clear that discussions about medicines did not involve patients when they took place in the ‘technical’ phase and were then not included in the summary to the patient.

#### Impact on study design

Finally, the process of making arrangements with potential lay observers also shaped the overall direction of the project. One of the potential lay observers suggested that he would particularly like to observe rounds where carers would be present, and so the research team took the decision to carry out some observations of evening drug administration rounds, which coincided with visiting hours to the ward.

### Challenges

#### Registration of lay observers as hospital volunteers

As noted above, lay observers needed to be registered as hospital volunteers in order to obtain a DBS check and to ensure that they were covered by appropriate indemnity insurance. Both lay and researcher interviewees reported considerable difficulty in this registration process. Interview data suggested that this was due to data collection being a different role to that typically carried out by hospital volunteers, and existing processes were not easily adaptable to this new role. The problems presented varied between the two trusts and with different lay observers. Interviews suggested that some of the lay observers had been quite upset by the process.

Well, the experience at [name of hospital 1] was okay, a trifle lengthy. The experience at [hospital 2] I thought was absolutely appalling. (Lay observer 2, discussing registration process)

It seemed to me that nobody seemed to know how to do it. They didn’t fall within the typical hospital volunteer profile, because they weren’t able to commit to doing so many hours a week in the hospital shop, or whatever it is that they're used to doing, and they were being recruited for a specific project. Nobody really knew how to deal with that, and it felt a bit like we were being passed from pillar to post, in terms of how we do it. It just took … a lot of effort and time. I think both for researchers and for the patients. I think it was a big commitment for everybody to try and make it happen. I think we all had to be quite motivated, otherwise it would have been very easy to just say, “You know what? This isn't worth the aggro”. (Researcher 4)

Similarly, when lay volunteers were asked about the training, all three lay observers found the volunteer training provided by the trusts to be long-winded or patronising. One of the researchers reported that there had also been delays in obtaining hospital identity cards for the lay observers. No unmet training needs were identified by the lay observers although the researchers identified a need for further training in research and information governance (see the next section).

#### Differing paradigms of information and research governance and codes of practice

Interview data suggested that lay observers without any previous experience as healthcare professionals may have had a different paradigm of information and research governance to the researchers. A common theme emerging from the interviews was the responsibility that researchers felt for ensuring information and research governance procedures were followed. In addition, one of the researchers reported that the healthcare professionals being observed had had similar concerns.

I guess one of the things is as well, you almost feel as though you’re responsible for them up on the wards so from a professionalism … perspective, it’s almost you’re on the line as well because they represent you because they’re with you. (Researcher 3)

Sometimes it would be initiated by the healthcare professional [being observed] to say, “Oh well are these lay observers allowed to be here … they would check that they had gone through the right means. And one consultant [senior doctor] said … “Have you got all the appropriate things to say you’re meant to be here?” Another consultant on another round said to us, “Oh we have really sensitive patients here, so I’d presume that you’re going to respect confidentiality”. So I … just wanted to reassure them yes they’ve gone through the .... appropriate checks. (Researcher 1)

The researchers involved in the observations reported that confidentiality and general code of practice were respected although they had had some minor concerns at times. However, these researchers also expressed concern the lay observers may sometimes have recorded data based on interactions with patients who had not consented to be included. The researchers had subsequently discussed how to approach this and excluded from analysis any data that may have been obtained from patients who had not consented. The interview data suggested that the lay observers did not share these concerns. In most cases where lay observers may have recorded data based on observation of patients who had not consented, they did not recall this at the time of interview, although the researchers reported that the lay observers’ observation notes suggested that they had been aware of it at the time. However, in one case, a lay observer did recall recording data from a patient who had not given consent; he or she had taken a more holistic view and expressed the opinion that collecting data from interesting cases, potentially of great value in informing future practice, overrode the requirement to obtain informed consent. The researchers ensured that data were discarded and excluded from analysis if they were not certain that informed consent had been given. These data suggest that while lay observers were aware of research governance procedures, they may have taken a more fluid or individual approach to their application, whereas the researchers felt bound to adhere strictly to the protocol approved by the ethics committee.

#### Timing

Lay involvement in data collection was suggested after ethics approval had initially been obtained for the IMPRESS study and very shortly before observations were due to start. This resulted in the need to submit a request for a substantial amendment to the ethics committee. Coupled with the delays in the registration process, this meant that the lay observers were not able to be involved in observations at the very start of the study. One of researchers expressed the view that if the lay observers had been involved with the earlier observations, these may have further informed her own later observations.

If we hadn’t had all the delays in getting them involved and we could have got them all registered earlier and they could have started their observations a bit earlier, I think it would have benefited the project because when I was doing the analysis of the project I did feel like some of the things they found were really interesting and I could have used it for my future observations …but… because it took so long to get their work through, that wasn’t really possible. (Researcher 2)

### Workload for researchers

While the researchers involved were very positive towards the lay observations, those involved in organising them also reported that it had increased their workload. This was due to the requirement for the researchers to invite consent from additional patients for the additional lay observations, orientate the lay observers to the ward and be available on the ward while they were doing their observations. There was also additional work related to the registration process and training. Coordinating observations was more complicated as the researchers had to find a time which was suitable for the healthcare professional being observed, the lay observer and the researcher supporting the lay observation. However, the lay observers and researchers noted that this process had run smoothly, and the researchers expressed the view that the value added to the research made the extra time worthwhile. However, they were of the view that this time would need to be taken account of in planning future lay involvement of this type.

With regards to when each lay observer came on to do their observations, orientating them to the wards, showing them the toilet, the facilities, and all that kind of thing, the introduction, that did take a while, but that can’t really be avoided. (Researcher 1)

There’s also logistical challenges about, scheduling observations on the wards is quite challenging, in terms of trying to link up with the healthcare professionals, and when it is a good day and a bad day on the ward? You can turn up and find that the consultant on that day isn’t happy for an observer to be there. Then, when we’ve also got to think about coordinating with lay observers, and according to our protocol, we always want to have one of the professional observers around on the ward at the same time. So it adds an additional logistical complexity to what is already logistically challenging in some ways. (Researcher 4)

#### Recruiting a representative group of lay observers

One of the researchers discussed the difficulties of recruiting a diverse group of lay observers. They reported that ideally they would have liked to recruit a diverse and more representative group of the public to carry out the observations. However, as noted above, recruitment had to rely on using personal contacts. All lay observers were over 55 and of a similar ethnic group. Some effort was made to widen the range of lay observers by approaching support groups for diseases likely to be found in younger disease groups, asking hospital consultants specialising in those diseases if they knew patients who may be interested and by asking university students. However, none of these approaches were successful.

Despite a lack of diversity in relation to age and ethnic group, one of the researchers was of the view that diversity in relation to different backgrounds and experiences of the lay observers greatly contributed to the quality of the data.

Another researcher raised the issue of ‘how “lay” is lay?’, suggesting that if lay people get involved in many research projects they may cease to become truly ‘lay’ and become more like healthcare professionals and less representative of the general public. The researcher who expressed this view did not think that this was the case in this particular study but that it was something to consider for future work. It should also be noted that one of our lay observers had had past experience as a physiotherapist.

Because—and I see it when we do the ethics committee, you know, you get a couple of new lay members and after a few years they all of a sudden are starting to talk all the jargon … . So, say for example, we had a couple of new lay members join us about three months ago and it’s actually really refreshing to hear what they’re saying on the applications … because I guess they haven’t been jaded by previous applications. So if you translate that then to a study, if you’ve got lay members who are involved a lot in lots of different research procedures, they will be almost experienced lay people. (Researcher 3)

#### Other challenges experienced by lay observers

Lay observers reported experiencing some challenges during the observations themselves, which were similar to those experienced by researchers when they carried out their observations. These included time spent hanging around due to not all patients consenting, ward rounds being rescheduled at the last minute, difficulties in remaining a passive observer when those being observed wanted to include the observer in conversation and the rounds observed only representing a snapshot of the patient’s full care.

Well sometimes the elderly patient in some cases [wanted] me to participate in the kind of discussion they were having, let’s say with the pharmacist or even a doctor, they wanted to call on me for more or less agree or disagree with what they were saying, hoping that perhaps because of my age I would know better than a young person what they were trying to say. … So the only challenge I had in some cases, not at all obviously was to keep very very mute, silent, control any body language except for being smiling obviously and not appear gruff or judgemental or anything, but to be really kind of statue in that sense. (Lay observer 2)

One of the researchers reported that a challenge specifically affecting lay observers was that local policy stated that they were not allowed to go into single bed rooms where infection control procedures were in place. This meant that they were more limited than the healthcare professional researchers who were allowed to enter these rooms provided they took appropriate infection control precautions.

### Lessons for future studies

Interviewees were asked about their view of lay involvement in conducting future research. All lay observers and researchers were positive about lay involvement in the future. Alongside this, both a lay observer and researcher expressed the view that while lay involvement in data collection had worked well in this context, this may not applicable to all types of studies. Both the lay observer and researcher were of the opinion that in this study, lay involvement in data collection was particularly relevant because the study focused on interactions between patients and healthcare professionals. However, there may be less of a place for lay involvement in observations of inter-professional issues which do not directly involve patients.

Well, I guess what we’re interested in, in this particular project, is about interactions between healthcare professionals, hospital patients, medicines. So the interactions with people is a key part of that. I could imagine that there would be many other studies where we’re actually interested in, I don’t know, interactions between pharmacists and doctors, which is perhaps slightly less relevant to get a public view on that, if those are interactions and communications that don’t really happen in front of the patient anyway. (Researcher 4)

In some projects actually the patient perhaps has to be limited to a consultative role, when the project is really aimed at healthcare professionals for them to introduce quality improvement programme[s]. (Lay observer 3)

Another recurring theme with regard to future projects was the need for proper planning in terms of lay involvement. This was both in terms of specific projects and overall coordination of patient and public involvement at hospital trusts. For future studies, it was suggested that it would be important to plan any lay involvement in data collection at an earlier stage so that resource could be allocated and the paperwork put in place in time. The majority of lay and researcher interviewees felt that improvement was also needed to streamline the registration and training processes. Suggestions included collaboration with the volunteer coordinating centre much further in advance for future projects, finding out whether it was possible to fast track DBS applications and completing all necessary procedures on one occasion rather than asking the lay observers to return on a large number of occasions.

How could my experience be improved for the future? Well, not having to go through all the inductions. (Lay observer 3)

The issue also arose as to how much training to give lay observers. The two researchers specifically involved in the observations were of the view that the role of the lay observers was to bring their own perspective and that while they wanted to provide training on research governance and ethical procedures, they had not wanted to provide much training on what to observe and record during the observations. A third researcher expressed uncertainty about the right level of training to include.

So I think if they were concentrating too hard on just like trying to find out – or just pick out the bits about medication or treatment, I think it might have meant that they were distracted. So I think it was probably a good thing actually that we just kind of left it up to them to just record what they felt they needed to record. (Researcher 1)

You could almost give them a whole training programme on how to find your way around a hospital, a ward, what communication structures, what theoretical framework filled that, to practically nothing, and then there’s a whole continuum in between.. I suppose what I don’t have a sense of is, where is the right place on that continuum for them and for the study? I think that something that, going forwards, we’ll probably need to have a better sense of, is to how much of that background information and training is needed to do the right job? (Researcher 4)

As noted above, the lay observers’ recollection was of the mandatory training provided by the trust rather than the more specific training the researchers had provided. However, the lay observers did report feeling supported by the researchers working with them.

And the investigators explained everything very clearly to the lay observers and basically facilitated the whole process extremely well. (Lay observer 2)

## Discussion

This study has demonstrated that involving lay people in data collection is one way of overcoming the problem of tokenistic patient involvement in research. Both the lay observers and the researchers working closely with them were of the opinion that they had contributed and added real value to the research. This perhaps provides a way forward in terms of breaking out of the self-fulfilling cycle of tokenism identified by Snape et al. [1]. In their systematic review, Domecq et al. [2] described the reported benefits of involving the lay public in research were that it improves patient enrolment in research studies and decreases subsequent attrition and aids in dissemination. These are all important roles, but the current study has demonstrated that the lay contribution can go far beyond this to adding a different perspective to the data collected and the ensuing research findings.

However, this increased involvement also brought challenges, such as maintaining the correct balance of the insider/outsider status of lay observers. Martin and Finn [4] have suggested that there may be a tension between achieving a true partnership rather than a consultative relationship between researchers and lay people and still keeping the lay input distinct from that of the researchers. Similarly, Ives et al. [3] discussed the paradox of the contribution of lay people needing to being outsiders to the system but needing to be trained to conduct research and become insiders. Ives et al. [3] suggest that this paradox may mean that it is not possible to give lay people the training they need to conduct research without compromising their ability to bringing a fresh perspective as an outsider and that their involvement should be limited to being consultative. The current study findings suggest that lay observers were involved in data collection in true partnership with the researchers, while still bringing their unique perspective to the research. The mechanism for this is unclear, but our researchers specifically sought to give the lay observers the minimum training necessary to fulfil their role. Yet, the findings also suggest that while lay members were given training on research governance procedures, they may not have been able to follow these fully without taking on a researcher/healthcare professional perspective of research ethics. It may be possible to find a way of maintaining the right balance of keeping the unique perspective of lay observers while still ensuring appropriate research governance; this is a subject for future research.

The need to recruit a diverse and representative range of lay people has been recognised [5]. Martin [5] discussed two models of representation, one of having statistical diversity in terms of demographic characteristics and the other embodied in the ability of the lay people to represent the needs of a diverse group of people, rather than being a diverse group in itself. In this study, it was easier to establish the latter as our lay observers included an experienced former inpatient who was therefore able to represent the patient experience and a former communications manager who had experience in making sure the needs of a diverse population were met. Researchers also need to take into account that lay people who are willing and available to take part in data collection are also likely to be a limited resource,

We have also identified the need for infrastructure to be developed to support this type of patient and public involvement In terms of training. INVOLVE [8] has developed resources for many lay involvement roles, but this does not extend to the specific training needed for data collection.

Some of these challenges have previously been described with relation to patient and public involvement in research, including patient frustration with lengthy processes of training and meetings, time restraints of researchers, extra time needed to complete research and additional funding needed [2]. However, introducing a greater level of involvement, as described here, intensifies these challenges and calls for the development of systems to minimise them. While additional time and resources may have alleviated these problems to some extent, we feel that an agreed system for negotiating access is needed.

A theoretical concern of patient and public involvement discussed in the literature is ‘scope creep’, i.e. that involving patients in the research may lead to irrelevant concerns and issues being suggested which would make the research unfeasible [2]. This was not a concern raised by the researchers or lay people involved in the present study. While the lay observers had a different emphasis in their observations, this only served to strengthen the findings.

On the basis of the positive results of lay involvement in data collection, we also subsequently took the decision to involve lay members in data analysis in a later part of the IMPRESS study, and all lay members are authors on the resulting publication (submitted for publication).

### Limitations of the study

This study was limited to interviews with a small number of lay observers and researchers who were involved in one research project. It is there unclear how generalisable the findings are. The researchers conducting the interviews were part of the research team rather than external to the study which meant that there was potential for social desirability bias. However, given the scarcity of studies addressing this issue, these findings can help inform future patient and public involvement in studies of this type. Future evaluations of this kind, with independent researchers, will be able to test cumulative validity for the study findings. Further research should also explore the views of hospital inpatients about data collection by lay observers; we did not explore this issue.

## Conclusions

Lay members of the public can add value by being involved in data collection for health services research projects by bringing their unique perspectives to the research. Maintaining the correct balance of not ‘professionalising’ lay people and giving them sufficient training to conduct research is a challenge. There is a need to address this further in future studies and to build infrastructure to support lay involvement in conducting research.

## Abbreviations

IMPRESS: Inpatient medication, patient relationships and electronic systems
NHS: National Health Service
